# The next forum for unraveling FDA off-label marketing rules: State and federal legislatures

**DOI:** 10.1371/journal.pmed.1002564

**Published:** 2018-05-08

**Authors:** Michael S. Sinha, Aaron S. Kesselheim

**Affiliations:** Program On Regulation, Therapeutics, And Law (PORTAL), Division of Pharmacoepidemiology and Pharmacoeconomics, Department of Medicine, Brigham and Women’s Hospital and Harvard Medical School, Boston, Massachusetts, United States of America

## Abstract

In a Guest Editorial, Aaron S. Kesselheim and Michael S. Sinha show how federal and state legislation to allow promotion of drugs for non-approved uses threatens to undermine the FDA's public health mission.

For the past half-century in the United States, it has been widely recognized that, even though physicians can legally prescribe drugs for indications that the Food and Drug Administration (FDA) had not approved (“off-label” uses), pharmaceutical manufacturers could not proactively promote their products for such uses. This rule, which arises from language in the federal Food, Drug, and Cosmetic Act (FDCA) [1] that gives the FDA its authority [2], has numerous public health rationales [3]. Of primary concern is the fact that off-label prescribing can carry substantial risks of both ineffectiveness and even harm for patients; permitting promotion for such a purpose could lead pharmaceutical manufacturers to flood the market with biased and/or incomplete information that can sway prescribing practices. Recognizing that some off-label communication could be permissible, the FDA has enumerated safe harbors for manufacturers: responding to unsolicited questions from physicians, distributing peer-reviewed article reprints discussing these uses, and sponsoring impartial continuing medical education courses. Nonetheless, in the past three decades, tens of billions of dollars in civil and criminal penalties have been paid by nearly all major pharmaceutical manufacturers for engaging in off-label promotion outside these circumscribed areas [4], in each case leading to problematic consequences for patients (such as from the widespread, promotion-driven use of antipsychotics in elderly patients with dementia) [5].

Off-label drug marketing came under scrutiny in a 2012 federal appeals court case, *US v*. *Caronia* [6]. In a split ruling, the judges extended protection for “commercial speech” under the First Amendment to off-label marketing statements made by a manufacturer’s sales representative. Viewing the current off-label marketing rules as infringements on “commercial speech,” the court postulated that other less restrictive alternatives—such as adding disclaimers to manufacturers’ statements—could achieve the same goals, despite evidence that such alternatives would be unworkable or unsuccessful in practice [7]. The *Caronia* court permitted manufacturers to engage in off-label promotion as long as it was “truthful and non-misleading,” a standard far less rigorous than the FDA’s current standards for determining a drug’s efficacy and safety for a particular indication. For example, a single study (such as a poorly controlled trial or badly done observational study) may have some “truth” to it about a drug’s possible effectiveness or safety but may badly misrepresent the totality of the evidence that the FDA considers, which may point in an opposite direction.

With the *Caronia* ruling only affecting the jurisdiction of a single appeals court, the FDA has continued to enforce its existing rules relating to manufacturer promotion, although it has enumerated additional safe harbors for off-label marketing. For example, in 2014, the FDA proposed two draft guidances [8,9] that would further loosen promotional rules by allowing manufacturers to distribute non–peer-reviewed clinical practice guidelines that describe off-label uses, as well as peer-reviewed studies that depict lower estimates of product risks than those determined by the FDA [10].

In the meantime, state and federal legislators have seized on the *Caronia* decision to propose statutory changes that would give wide latitude to manufacturers engaging in off-label promotion. Under the 21st Century Cures Act of 2016, manufacturers are now allowed to provide healthcare economic information about off-label drug uses to formulary committees or other similar entities that help insurers make drug coverage decisions [11]. Such groups have more resources than individual physicians to critically evaluate such claims, although there is still a substantial range in their sophistication across the US market. In March 2017, Arizona passed the Free Speech in Medicine Act, which explicitly permits manufacturers to communicate with physicians and other prescribers about off-label uses [12]. The legislation would be unlikely to survive a legal challenge based on preemption by the federal FDCA. However, invoking such a challenge may be one of the goals of the law’s key proponent, the Goldwater Institute, in its effort to limit the FDA’s ability to regulate off-label promotion [13].

Two bills were also introduced in the US House of Representatives in 2017 to expand the permitted range of off-label promotion. The Medical Product Communications Act [14] seeks to create a new safe harbor for “scientific exchange” with prescribers relating to off-label uses as long as the communication “is not advertising or otherwise promotional in nature,” the communication is supported by “competent and reliable scientific evidence,” and manufacturers provide “appropriate contextual information.” This bill leverages the fact that manufacturers’ sales representatives often engage physicians in “scientific exchange” to meet their promotional goals, and in doing so, the legislation would allow the dissemination of clinical data that would not necessarily meet the FDA’s substantial evidence of efficacy standard, resulting in the communication of biased, incomplete, or inaccurate studies. The Pharmaceutical Information Exchange Act [15] attempts to expand the range of insurance coverage–related discussions established in the 21st Century Cures Act by allowing the manufacturers to present information about unapproved uses to formulary or technology review committees that it “anticipates could be sufficient” to support future FDA approval of such unapproved use; this could include preclinical data. Both bills would require manufacturers to include disclaimers that the FDA had not approved the information, but disclaimers currently available in the context of non–FDA-approved promotional claims relating to nutritional supplements have not been demonstrated to work [7].

Buoyed by a narrow victory in one appeals court, advocates have turned to state and federal legislatures to unravel current FDA rules relating to off-label promotion. But these rules are essential for the ability of the FDA to fulfill its public health mission by defining what uses of drugs have benefits that outweigh their risks versus those that lack sufficient evidence to warrant such use. These distinctions are crucial for individual physicians—who do not have the time or expertise to perform the same critical data evaluation conducted by the scores of highly trained scientists at the FDA—and for patients, who could be exposed to more non–evidence-based and potentially dangerous off-label uses of high-cost drugs.

## Abbreviations

FDA: Food and Drug Administration
FDCA: Food, Drug, and Cosmetic Act
